# What place for radiographers? The appropriateness of preliminary image evaluation in New Zealand emergency departments

**DOI:** 10.1002/jmrs.817

**Published:** 2024-08-26

**Authors:** Ryan Walklin

**Affiliations:** ^1^ Radiology Department Taranaki Base Hospital, Te Whatu Ora Taranaki New Plymouth New Zealand

## Abstract

Radiology services in New Zealand are under significant pressure. Preliminary image evaluation (PIE) by radiographers can have a significant positive impact on patient care in this constrained environment and should be supported.

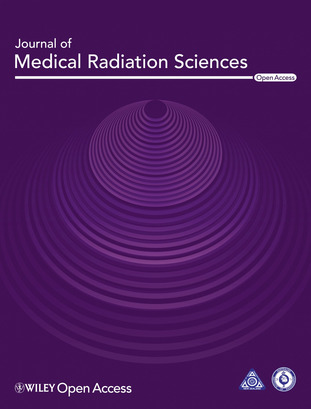

## Introduction

Public radiology services in Aotearoa New Zealand are under significant pressure, with ever‐increasing demand across all modalities. This increase is driven by an ageing population, limitations in primary and secondary care translating to increased imaging utilisation and ever‐increasing complexity of imaging modalities.^1^

Likewise, public hospital emergency departments are suffering from increasing demand and an increasingly complex workload. Patients with chronic problems increasingly present *in extremis* with undiagnosed and advanced malignancies, or with acute surgical, medical or mental health emergencies. Often these patients have not been able to access timely primary or secondary care before their condition deteriorates. This overburdens acute services not configured or resourced to deal with them and displaces elective and planned care from public hospitals.

## Radiology and Te Whatu Ora

The creation of a new national health system for New Zealand, Health New Zealand Te Whatu Ora, based on the recommendations of the 2020 Health and Disability System Review,^2^ is intended to reduce variation in care across New Zealand and improve the delivery of both primary and secondary care. However, the nature and extent of the reforms have been criticised, and it remains to be seen whether they will deliver on their promise of Pae Ora, “Healthy Futures.” Meanwhile, publicly funded radiology services continue to struggle with under‐resourcing, ageing equipment, unfit and obsolete digital systems and a critical workforce shortage.

Short‐term mitigation of these difficulties by outsourcing imaging and reporting to private providers reduces waitlists but is inequitable due to the distribution and capability of private facilities, catering predominantly to ambulatory care in larger centres. It diverts resources and capacity away from the public sector, by luring radiographers and radiologists away from public employment with improved remuneration and working conditions. The cost of this outsourcing is also being questioned by funders, with weakened public services not in a position to respond effectively.

A National Clinical Network for Radiology was established by Te Whatu Ora in early 2024 (as a continuation of its predecessor the National Radiology Advisory Group) to address these challenges. Many solutions have been proposed by this group, including the use of a pathway‐based approach to imaging, increasing radiology registrar training capacity, recruitment of qualified radiologists from overseas, AI tools, and increasing access to imaging for community referrers.

However, none of these initiatives have yet been deployed widely as a robust solution for mitigating the challenge of increasing demand and constrained supply and ignores radiographers, a highly competent workforce already working side by side with radiologists.

## What Place for Radiographers?

Role extension for radiographers has previously been considered by the comparable health systems in the United Kingdom (UK) and Australia. In the UK, formal radiographer reporting has been in place for some years,^3^ and a framework for reporting musculoskeletal trauma films is jointly published by the College of Radiographers and Royal College of Radiologists.^4^ Undoubtedly, the popularity of this practice is in part related to the UK radiologist workforce crisis, which continues to deepen.^5^

In Australia, role extension to reporting for radiographers has been more controversial. The Royal Australian and New Zealand College of Radiologists (RANZCR) published a position statement in 2018 strongly against radiographer scope extension, noting, ‘RANZCR does not support the substitution of clinical radiologists by radiographers for the sake of career development or to reduce costs, particularly at a time when there are sufficient clinical radiologists to allow for reporting in Australia’.^6^ This stance was not well received by the Australian radiographer community.^7^

Although RANZCR is responsible for radiology training in both Australia and New Zealand, this document focused almost entirely on the Australian context. It would be incorrect to state that there are sufficient clinical radiologists to allow for reporting in New Zealand in 2024. It is fair to note, as RANZCR does, that there are differences in training between UK reporting radiographers and consultant radiologists. There are also significant medicolegal and other challenges related to formal reporting by radiographers in an Australasian context, and additional financial implications relating to Medicare reimbursement in Australia.

The position statement went on to conflate radiographer formal reporting and preliminary image review and opposed both, which is unhelpful given that “red dots” on X‐ray packets have been long‐standing practice throughout Australia and New Zealand, and welcomed by many a tired emergency registrar at 3 am. Conversely, it is routine in Australasian teaching departments to allow radiology registrars on call after as little as 6 months of formal training, supported by a consultant radiologist, and for registrars to issue preliminary or interim reports. These reports are reviewed by consultants before sign‐off, however, they do guide acute management, with an acknowledgement by the service that they are subject to change.

It is therefore hard to justify why our radiographer colleagues cannot reach a similar level of supervised expertise, and thankfully, the practice has gained wider Australian acceptance and demonstrated benefit in the years since.^8^

## The Value of Preliminary Image Evaluation

Basic and advanced radiology services are increasingly utilised in emergency departments, with computed tomography (CT), magnetic resonance imaging (MRI) and ultrasound all commonly requested directly from emergency physicians. There has also been significant role expansion in other allied health professions, with nurse practitioners, physiotherapists and even paramedics requesting acute trauma imaging.

A robust training and credentialing program has been set up by our department (nurse‐initiated imaging or NIX) to ensure these practitioners are supported to order safely, and it is also incumbent on services to support them and their patients with rapid and reliable interpretations. It is simply not possible in the current New Zealand context to provide this interpretation via a formal radiologist report for every X‐ray at the time it is taken, and the preliminary image evaluations (PIEs) completed by radiographers in our department have proven invaluable in routine practice, to say nothing of the findings which would have otherwise been missed, even by consultant radiologists.^9^

As our local experience has demonstrated, the interpretive performance of radiographers can approach and in some cases surpass radiologists. However, this is perhaps not the comparison that we should be making. Given a backlog of unreported X‐rays for which only an emergency clinician (which may be a nurse practitioner or a junior doctor) has given an opinion, there is a real risk that treatment is guided by an interpretation that has significantly lower accuracy than a PIE‐trained radiographer. There are also robust medicolegal arguments for the practice of PIE, informed by analysis of patient harm incidents where a radiographer's preliminary interpretation may have prevented an adverse outcome.

Less easily measured but equally important benefits have also been noted. Increased job satisfaction for radiographers and strengthened working relationships between radiographers and radiologists have resulted from the collaboration enabled by the PIE process as well as a significant uplift in image quality. The ability of radiographers to detect abnormalities on their X‐rays is derived directly from their expertise in technical image evaluation, which has been recognised by my colleagues locally as well as reported in the literature.^10^

## Conclusion

Publicly delivered radiology in New Zealand is at something of a crossroads, with numerous threats to its viability at a time when its value to patient care has never been higher. At the same time, the cost of delivering radiology is increasing, exacerbated by an increasing reliance on outsourcing, and this is being challenged by funders.

Preliminary image evaluation performed by radiographers cannot mitigate this alone but has a significant positive impact on acute patient care in the emergency department, contributes to increased patient safety and quality, and aids job satisfaction and retention of our radiographers. We should not be afraid of the implications of increasing radiographer scope; instead, we should be concerned with the harm to our patients if we do not.

## Conflict of Interest

The authors declare no conflict of interest.

## Funding Information

No external funds were provided.

## Data Availability

Data sharing is not applicable to this article as no new data were created or analyzed in this study.
